# When Postoperative Inflammation Isn’t Benign: Thyroid Eye Disease Following Cataract Surgery

**DOI:** 10.7759/cureus.95196

**Published:** 2025-10-22

**Authors:** Yilynn Chiew, Khay Wei Poh

**Affiliations:** 1 Ophthalmology, Hospital Shah Alam, Shah Alam, MYS

**Keywords:** cataract surgery, diplopia, graves’ orbitopathy, orbital inflammation, orbitopathy, postoperative complication, proptosis, thyroid eye disease

## Abstract

Thyroid eye disease (TED) is an autoimmune inflammatory disorder of the orbital tissues, most commonly associated with Graves’ hyperthyroidism. Although it typically occurs in the presence of thyroid dysfunction, TED may also arise in euthyroid or subclinical states. Its onset after ocular surgery is uncommon and may mimic routine postoperative inflammation, delaying diagnosis. A 67-year-old Chinese-Malaysian man developed eyelid swelling, redness, and blurred vision two weeks after uncomplicated left-eye cataract surgery. He was initially treated for common postoperative complications, but his condition progressively worsened, with visual acuity declining from 6/12 (6/9 with pinhole preoperatively) to 6/30 without pinhole improvement over six months, accompanied by proptosis, diplopia, and optic nerve dysfunction. Laboratory tests revealed subclinical hyperthyroidism, and orbital contrast-enhanced computed tomography confirmed tendon-sparing enlargement of all extraocular muscles, consistent with sight-threatening TED. He received intravenous methylprednisolone, mycophenolate mofetil, and carbimazole, followed by urgent medial wall decompression due to worsening optic neuropathy, with subsequent visual improvement. TED following cataract surgery is rare but underscores the importance of considering atypical postoperative courses. Early recognition and adherence to guideline-based management are crucial to preserve vision.

## Introduction

Thyroid eye disease (TED), also known as Graves’ orbitopathy, is a complex autoimmune condition affecting the orbital soft tissues, extraocular muscles, and orbital fat [1]. It is most commonly associated with Graves’ hyperthyroidism, but can also occur in euthyroid or hypothyroid patients, making its diagnosis challenging [2]. The pathogenesis involves autoimmune reactions targeting shared antigens, such as the thyroid-stimulating hormone (TSH) receptor, on thyroid follicular cells and orbital fibroblasts, leading to inflammation, glycosaminoglycan accumulation, and adipogenesis within the orbit [3].

The presentation of TED can vary widely, from mild eyelid retraction and proptosis to severe, sight-threatening complications such as compressive optic neuropathy [4]. Diagnosing TED can be particularly difficult when symptoms are subtle, atypical, or occur in individuals without a known history of thyroid dysfunction [5,6]. Cases of insidious or noninflammatory TED have been reported, where typical inflammatory signs are minimal, contributing to diagnostic delay [5].

A growing body of literature has examined the relationship between ophthalmic surgery and the onset or exacerbation of TED. While some studies have focused on refractive prediction errors after cataract surgery in patients already diagnosed with TED [7], others have reported the emergence of previously unsuspected TED following cataract or other periocular surgeries [6,8,9]. One study found that 14% of patients presenting with strabismus after cataract surgery were subsequently found to have previously unsuspected TED, with some having a history of dysthyroidism but no diplopia prior to surgery [8]. This suggests that surgery may act as an unmasking event or even a trigger for the manifestation of the disease [6]. Differentiating TED from common postoperative inflammatory complications, such as sterile inflammation, orbital pseudotumor, or other orbital inflammatory disorders, presents a significant diagnostic challenge [10,11].

We present the case of a 67-year-old Chinese-Malaysian man who developed significant ocular symptoms following uneventful cataract surgery, which were initially misattributed to common postoperative complications. The delayed recognition of sight-threatening TED in this context underscores the importance of maintaining a high index of suspicion for TED in patients presenting with persistent or atypical orbital inflammation after cataract surgery, even in the absence of known thyroid disease.

## Case presentation

A 67-year-old Chinese-Malaysian man, a nonsmoker with type 2 diabetes mellitus, hypertension, and essential thrombocythemia, presented with a three-day history of left-sided eyelid swelling, redness, discomfort, and blurred vision. These symptoms began two weeks after an uneventful left-eye cataract surgery.

He had a known history of primary angle-closure glaucoma for which bilateral peripheral iridotomies had been performed, and his intraocular pressure (IOP) was well controlled with topical timolol 0.5% twice daily. There was no family history of ocular diseases, including glaucoma, TED, or thyroid dysfunction.

**Figure 1:**
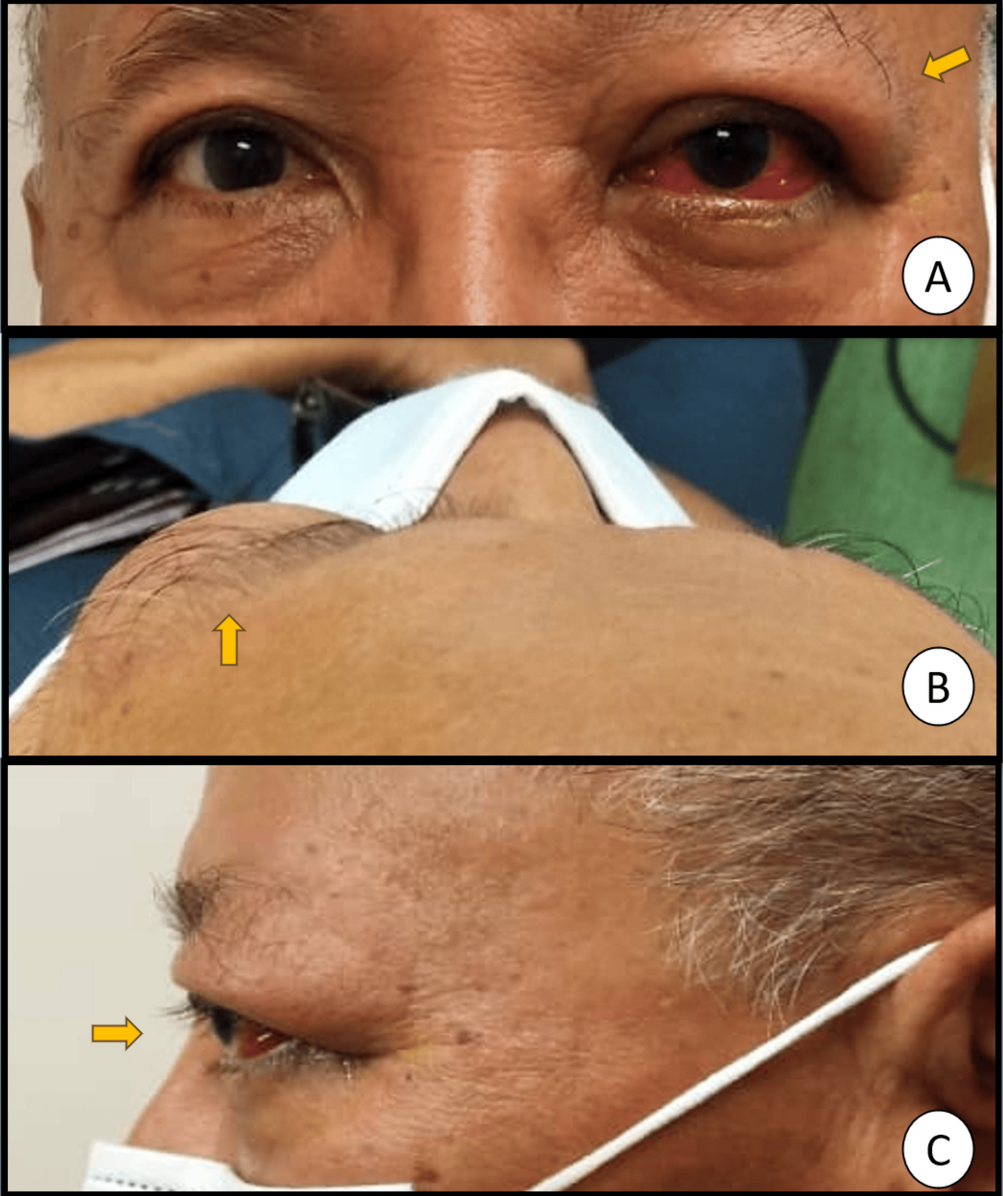
Clinical photographs showing proptosis of the left eye. (A) Frontal view demonstrating left eye proptosis with conjunctival injection. (B) Superior (bird’s-eye) view highlighting forward displacement of the left globe. (C) Lateral view confirming asymmetry and anterior protrusion of the left eye. Arrows indicate the proptotic left eye. Photographs were taken at the Ophthalmology Department, Hospital Shah Alam, Malaysia.

Despite multiple ophthalmologic consultations, his symptoms persisted for six months. He was initially managed as having prolonged postoperative inflammation, recurrent corneal abrasion, dry eyes, and possible hypersensitivity to topical steroid eye drops, but his condition progressively worsened. Over this period, visual acuity declined from 6/12 (6/9 with pinhole preoperatively) to 6/30 with no pinhole improvement, accompanied by increasing left-sided proptosis (Figure 1), restricted extraocular movements with diplopia, and gaze-evoked pain.

Optic nerve function testing in the left eye revealed reduced brightness appreciation to 60%, red desaturation to 10%, and Ishihara color vision 14/17, with a Bjerrum screen demonstrating an enlarged blind spot. The right eye showed full optic nerve function.

Proptosis measured with Hertel’s exophthalmometer was 18 mm in the left eye and 10 mm in the right (base 105 mm). Extraocular motility testing showed left-eye restriction of abduction (-3), adduction (-2), and depression (-1), with full elevation. Pain was elicited on eye movements, and diplopia was noted in left lateral gaze.

**Figure 2:**
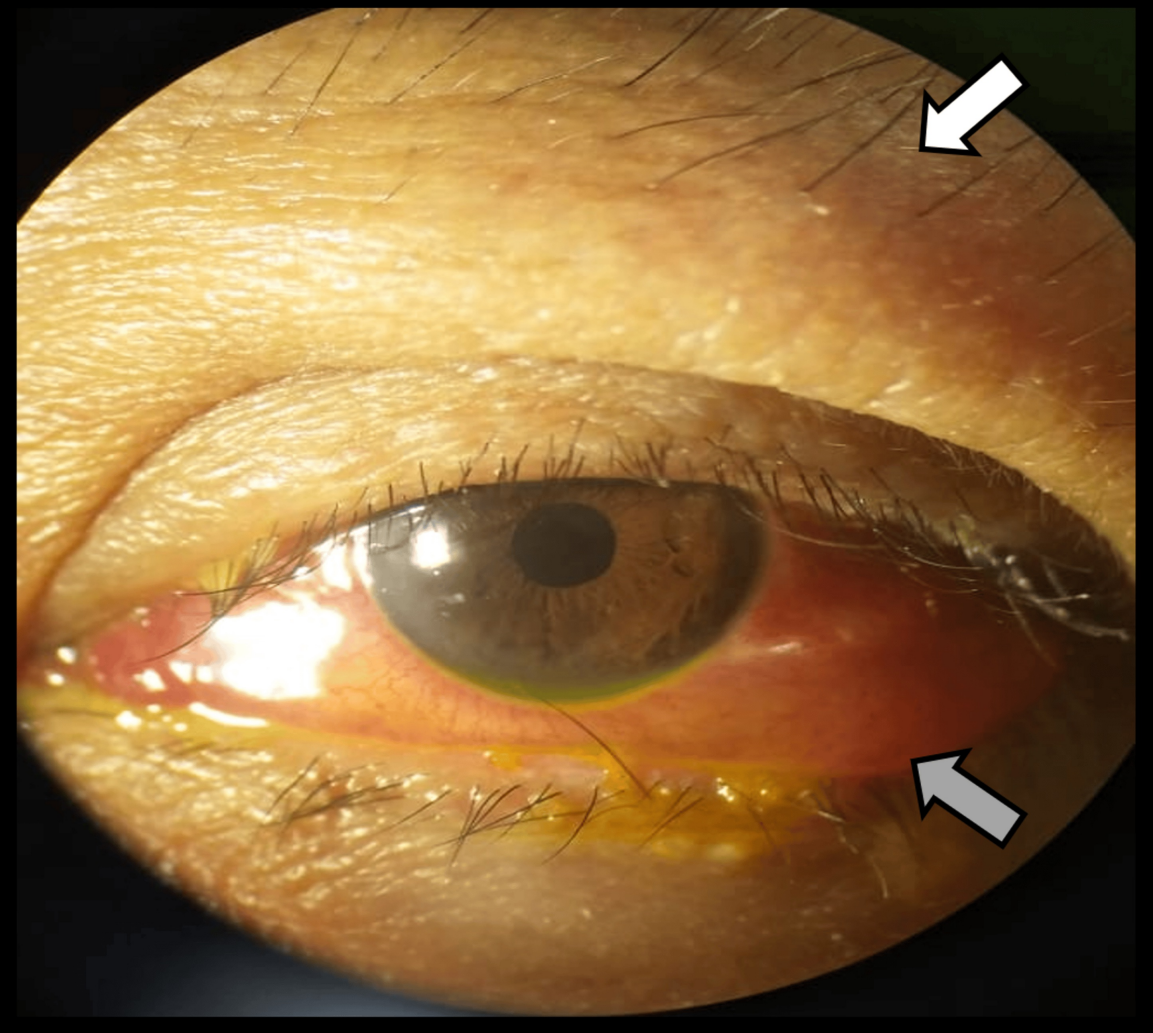
Clinical photograph showing swollen, erythematous, and non-tender left eyelids (white arrow) with generalized conjunctival injection and chemosis (grey arrow), findings consistent with active thyroid eye disease. Photographs were taken at the Ophthalmology Department, Hospital Shah Alam, Malaysia.

Anterior segment examination revealed swollen, erythematous, non-tender left eyelids with generalized conjunctival injection, chemosis, and a swollen caruncle (Figure 2). No lid retraction or lid lag was observed. Corneal findings included punctate epithelial erosions, minimal Descemet’s striae, and occasional anterior chamber cells, likely secondary to postoperative surface inflammation. IOP was 20 mm Hg bilaterally.

**Figure 3:**
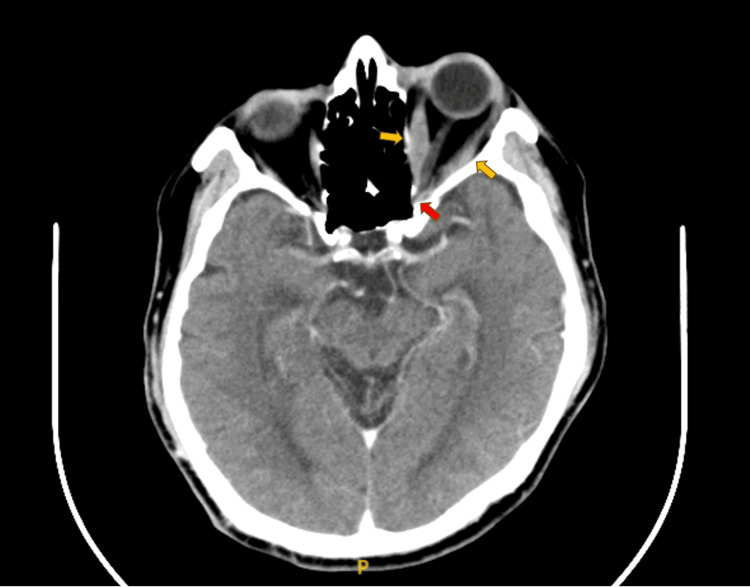
Axial contrast-enhanced CT (CECT) of the orbits showing tendon-sparing enlargement of the left medial and lateral rectus muscles (yellow arrows) with apical crowding and compression of the optic nerve (red arrow). Image obtained at the Radiology Department, Hospital Shah Alam, Malaysia.

Dilated fundus examination of the left eye showed a pink optic disc with a cup-to-disc ratio of 0.8 (unchanged from preoperative assessment) without disc swelling. The macula exhibited an epiretinal membrane, and the retina appeared flat with visible choroidal folds. The right-eye fundus showed a pink optic disc with a cup-to-disc ratio of 0.8 and was otherwise unremarkable. B-scan ultrasonography of the left eye revealed a clear vitreous, flat retina, and no evidence of intraocular or orbital mass, scleral thickening, or T-sign. Contrast-enhanced computed tomography (CECT) of the orbits demonstrated left-sided proptosis with tendon-sparing enlargement of all extraocular muscles, most prominently the inferior and medial recti, with crowding of the orbital apex and mild compression of the optic nerve. The right orbit appeared normal (Figure 3).

Additional systemic workup was performed to exclude infectious and inflammatory causes. Tuberculosis screening showed a negative Mantoux test and a normal chest radiograph, with an erythrocyte sedimentation rate (ESR) of 3 mm/hour (reference < 20 mm/hour). Full blood count demonstrated thrombocytosis, consistent with the patient’s known essential thrombocythemia, and peripheral blood film confirmed thrombocytosis without evidence of disease progression.

Thyroid function testing at the time of diagnosis demonstrated suppressed TSH 0.276 mIU/L (reference 0.55-4.78) and free thyroxine (FT4) 20.12 pmol/L (reference 11.5-22.7), consistent with subclinical hyperthyroidism. Autoantibody testing revealed antithyroglobulin antibody < 0.9 IU/mL (normal), antithyroid peroxidase antibody 8.78 IU/mL (reference 0-9), and anti-TSH receptor antibody (TRAb) 3.67 IU/L (reference < 1.75), confirming autoimmune thyroid activation. Ultrasound of the thyroid and neck showed diffuse thyroiditis with bilateral nodules (TI-RADS 3 and 4). Based on these findings, the endocrinology team diagnosed Graves’ disease and initiated carbimazole 10 mg once daily, started concurrently with ocular immunosuppressive therapy (within the same week as the initial intravenous methylprednisolone course). Subsequent follow-up showed biochemical stabilization, with thyroid function remaining within the euthyroid range under continued carbimazole therapy.

The patient was co-managed with the oculoplastic and endocrinology teams. Based on the EUGOGO 2021 classification [4], the disease was categorized as sight-threatening TED due to dysthyroid optic neuropathy (DON), evidenced by a decline in visual acuity (6/12 → 6/30), the presence of a relative afferent pupillary defect (RAPD), reduced brightness appreciation (60%), marked red desaturation (10%), impaired color vision (Ishihara 14/17), and a progressively enlarged blind spot on Bjerrum testing, together with apical crowding on CECT imaging.

At diagnosis, the Clinical Activity Score (CAS) [4] was 7/7, indicating spontaneous and gaze-evoked orbital pain, eyelid swelling and erythema, conjunctival redness, chemosis, and inflammation of the caruncle.

Initial therapy consisted of intravenous methylprednisolone (IVMP) 1 g/day for three consecutive days (cumulative dose 3 g), together with oral mycophenolate mofetil 1 g twice daily, in accordance with EUGOGO recommendations for active, sight-threatening disease.

Following this course, visual acuity improved to 6/30 (pinhole 6/20), with corresponding improvement in brightness appreciation (80%), red desaturation (80%), and Ishihara color vision (17/17). RAPD improved to grade 1. Eyelid erythema, conjunctival injection, and chemosis resolved, and CAS decreased to 2/10 (eyelid swelling and gaze-evoked pain).

He was maintained on weekly IVMP 500 mg for six weeks (cumulative 6 g) and planned for a second cycle of IVMP 250 mg weekly. However, midway through the second cycle (cumulative 6.25 g), his condition worsened: visual acuity declined to 6/40 (pinhole 6/30) with RAPD grade 2, brightness appreciation 70%, red desaturation 80%, Ishihara 12/17, and an enlarged blind spot. Extraocular motility deteriorated (abduction -3, adduction -2, depression -1), and Hertel exophthalmometry increased from 18 mm to 19 mm. Recurrent findings included eyelid swelling, conjunctival redness, and caruncle edema, giving CAS 4/10 (eyelid swelling, conjunctival redness, vision drop ≥ 1 Snellen line, and reduction in uniocular excursion > 8°). Repeat CECT demonstrated persistent extraocular muscle enlargement with increased compression of the left optic nerve.

Given the worsening active, sight-threatening TED (DON), a second course of IVMP 1 g/day for three days (cumulative 9.25 g) was administered, followed by left endoscopic medial orbital wall decompression with septoplasty, uncinectomy, maxillary antrostomy, ethmoidectomy, and sphenoidotomy performed under the otolaryngology team.

Post-decompression, visual acuity improved to 6/20 (pinhole 6/15), with stable RAPD grade 2, brightness and red desaturation 80%, Ishihara 16/17, and a stable enlarged blind spot. Lid fullness decreased (CAS 1/10). Extraocular motility improved (abduction/adduction -2; depression/elevation -1), and Hertel exophthalmometry remained 19 mm.

He subsequently completed IVMP 500 mg weekly for six weeks, followed by 250 mg weekly for another six weeks (final cumulative dose 13.75 g). Throughout therapy, blood pressure, blood glucose, and liver enzymes were monitored weekly, with cardiac and vital-sign monitoring during infusions. Gastroprotection and calcium supplementation were provided, and the endocrinology team co-managed steroid tapering and adrenal support. The patient has since remained clinically stable on maintenance oral mycophenolate mofetil 1 g twice daily. 

A summary of the patient’s disease course is presented in Table 1, outlining the sequential changes in visual acuity, proptosis (Hertel measurements), extraocular motility, optic nerve function, disease activity (CAS), and relevant laboratory and imaging findings at key time points, from pre-cataract baseline through postoperative onset, peak disease, corticosteroid therapy, orbital decompression, and final follow-up.

**Table 1:** Clinical timeline and key parameters before and after therapy. *Intraocular pressure remained stable in primary gaze and upgaze bilaterally throughout the disease course.* CAS: Clinical activity score; TRAb: thyrotropin receptor antibody; TSH: thyroid-stimulating hormone; TFT: thyroid function test; CT: computed tomography; ONFT: optic nerve function tests; RE: right eye; LE: left eye; EOM: extraocular muscle; MMF: mycophenolate mofetil; IVMP: intravenous methylprednisolone; DON: dysthyroid optic neuropathy; TED: thyroid eye disease.

Time Point	Visual Acuity (VA)	Hertel (mm, base 105)	Extraocular Motility	Optic Nerve Function Tests (ONFT)	Clinical Activity Score	Other Investigations	Cumulative IVMP Dose (g)	Key Findings/Management
Pre-cataract (baseline)	6/12 ph 6/9	-	Full	RAPD negative	-	-	-	PACG on timolol; no orbital signs
2 weeks to 6 months post-op (onset)	Ranging 6/15 to 6/60	-	Full	Not done	-	-	-	Mild lid swelling, redness, and blurring of vision; multiple consultations; treated as common post-operative complications
6 months post-op (at peak, pre-IVMP)	6/30 (no ph improvement)	RE 10, LE 18	Abd –3, Add –2, Dep –1	RAPD grade 1; Brightness: 60%; Red desaturation: 10%; Ishihara: 14/17 Bjerrum: enlarged blind spot	7/7*	TSH: 0.276 ↓ (0.55–4.78); FT4: 20.12 (11.5–22.7); TRAb: 3.67 ↑ (<1.75); CT: left eye proptosis, Optic nerve compression, tendon sparing enlargement of all extraocular muscles	0	DON confirmed; TED diagnosed (sight-threatening); start IVMP 1 g × 3 days + MMF 1g BD + carbimazole 10mg OD
Post-IVMP (after 1g OD X 3 days)	6/30 ph 6/20	RE 10, LE 18	Abd –2, Add –2, Dep –1	RAPD: +1; Brightness: 80%; Red desaturation: 80%; Ishihara: 17/17	2/10	-	3	Improved optic nerve function and soft tissue inflammation. Proceed with IVMP 500mg weekly X 6 weeks and then 250mg weekly X 6 weeks
During the second cycle (cumulative 6.25 g)	6/40 ph 6/30	RE 12, LE 19	Abd –3, Add –2, Dep –1	RAPD: +2; Brightness: 70%; Red desaturation: 80%; Ishihara: 12/17	4/10	CT: Persistent EOM enlargement, increased optic nerve compression	6.25	Recurrence of DON features with increased CAS; repeat IVMP 1g X 3 days and orbital wall decompression planned
Post-decompression cumulative 9.25 g cumulative IVMP)	6/20 ph 6/15	RE 12, LE 19	Abd/Add –2, Dep/Elev –1	RAPD: +2; Brightness: 80%; Red desaturation: 80%; Ishihara: 16/17	1/10	-	9.25	Improved vision and motility; stable post-surgery
Follow-up (after full course, 13.75 g cumulative IVMP)	6/20 ph 6/15	RE 13, LE 19	Stable	Stable	1/10	-	13.75	Stable TED on MMF 1 g BD; euthyroid on carbimazole 10 mg OD

## Discussion

This case illustrates the diagnostic complexities associated with insidious TED, particularly when its onset temporally coincides with common ophthalmic procedures such as cataract surgery [6]. The patient’s early presentation with eyelid swelling, redness, and discomfort two weeks after surgery closely resembled typical postoperative inflammation or complications such as corneal abrasion or drug hypersensitivity [10]. This resulted in a significant delay in diagnosis, with symptoms persisting for nearly six months before classical signs of TED, including proptosis, restrictive extraocular movements, and diplopia, became evident. Such delays are not uncommon in cases where TED masquerades as other conditions, especially when orbital findings are subtle or initially absent [6,12].

The insidious course in this patient was further highlighted by the presence of subclinical thyroid dysfunction rather than overt disease at the time of diagnosis [2]. While TED most often occurs in hyperthyroid patients, it can also develop in hypothyroid, euthyroid, or subclinical hyperthyroid and hypothyroid states, making recognition difficult in the absence of systemic signs [2]. The absence of preceding thyroid dysfunction symptoms, coupled with the postoperative context, compounded the diagnostic challenge. Imaging ultimately provided clarity, with the characteristic tendon-sparing enlargement of the extraocular muscles on orbital CT distinguishing TED from other orbital inflammatory disorders such as idiopathic orbital inflammation or orbital pseudotumor [11].

Although the mechanism by which cataract surgery might influence TED remains unclear, surgical trauma and the resulting inflammatory milieu may unmask subclinical disease or trigger clinical manifestations in predisposed individuals [6,9]. Ophthalmic surgeries can induce inflammatory responses [13], and in predisposed individuals, this could theoretically exacerbate an underlying autoimmune process or render previously subclinical TED clinically apparent.

This case contributes to the body of evidence suggesting that surgical stress may play a role in the clinical emergence of TED [6,8]. The management of sight-threatening TED requires a multidisciplinary approach. In this case, high-dose pulsed intravenous methylprednisolone (1 g/day for three days) was initiated, followed by weekly intravenous therapy and the addition of mycophenolate mofetil and carbimazole. Owing to progressive optic nerve dysfunction, urgent medial orbital wall decompression was performed, resulting in clinical improvement. The patient subsequently continued on weekly intravenous methylprednisolone, later tapered to oral prednisolone. This regimen is consistent with EUGOGO guidelines, which recommend intravenous glucocorticoids as first-line therapy for active, severe TED and urgent surgical decompression for DON not responding to steroids [4].

This case underscores the importance of maintaining a high index of suspicion for TED in patients presenting with persistent or atypical orbital inflammation and binocular diplopia after cataract surgery, even in the absence of systemic thyroid disease. Early recognition and adherence to guideline-directed therapy are crucial to preserving vision and optimizing outcomes [6].

## Conclusions

TED should be considered in patients presenting with persistent or atypical orbital inflammation and diplopia following cataract surgery, even without known thyroid dysfunction. This case underscores the potential for TED to mimic routine postoperative inflammation, leading to diagnostic delay. Maintaining a high index of suspicion and pursuing early imaging and multidisciplinary evaluation are crucial for timely diagnosis and preservation of vision.
